# Variability of the modified Balance Error Scoring System at baseline using objective and subjective balance measures

**DOI:** 10.2217/cnc.15.5

**Published:** 2015-08-06

**Authors:** Amaal J Starling, Danielle F Leong, Jamie M Bogle, Bert B Vargas

**Affiliations:** 1 Department of Neurology, Mayo Clinic Arizona, 5777 E Mayo Blvd, Phoenix, AZ 85054, USA; 2 King–Devick Test, Inc., 2 Mid America Plaza, Suite 110, Oakbrook Terrace, IL 60181, USA; 3 Department of Otorhinolaryngology, Mayo Clinic Arizona, 13400 E Shea Blvd, Scottsdale, AZ 85260, USA; 4 Department of Neurology, Mayo Clinic Arizona, 5777 E Mayo Blvd, Phoenix, AZ 85054, USA

**Keywords:** athlete, baseline, concussion evaluation, modified Balance Error Scoring System, postural control, sports concussion, standing balance

## Abstract

**Aim:**

To investigate preseason modified Balance Error Scoring System (mBESS) performance in a collegiate football cohort; to compare scores to an objective mobile balance measurement tool.

**Materials & methods:**

Eighty-two athletes completed simultaneous balance testing using mBESS and the King–Devick Balance Test, an objective balance measurement tool. Errors on mBESS and objective measurements in the double-leg, single-leg (SS) and tandem stances were compared.

**Results:**

Mean mBESS error score was 7.23 ± 4.65. The SS accounted for 74% of errors and 21% of athletes demonstrated the maximum error score. There was no significant correlation between mBESS score and objective balance score.

**Conclusion:**

The high variability and large number of errors in the SS raises concerns over the utility of the SS in identifying suspected concussion.

An estimated 1.6–3.8 million sports-related concussions occur in the USA every year [1]. The true incidence of concussion is felt to be much higher given the fact that countless individuals with concussion are either undiagnosed or simply fail to present to a healthcare provider. Several symptoms are typically associated with acute concussion [2]. These include subjective complaints of dizziness and imbalance, which are reported by approximately 40% of athletes in the hyperacute to acute stages. Also objective abnormalities on formal balance testing that can be identified weeks after injury [2]. Balance requires integration of the visual, vestibular and somatosensory systems. Following concussion, individuals often demonstrate impairments in the perception and processing of vestibular system information, especially when visual and somatosensory inputs are disrupted or altered, and often demonstrate a visual preference for maintaining balance [3]. Standing balance abnormalities are most apparent in the first few days after concussion as measured with static balance methods [4]. The reason for reduced balance postconcussion is not always apparent, as measures of peripheral vestibular dysfunction are often within normal limits, and diagnostic measures of central vestibulopathy are not often available [5]. For patients with central vestibulopathy (e.g., vestibular migraine), it has been hypothesized that the close connections between the vestibular nuclei and the oculomotor nuclei may lead to reduced visual integration [6–9]. Because balance often relies heavily on visual information following concussion, conflicts between sensory systems are frequently unable to be resolved appropriately, leading to increased presentation of imbalance and fall risk [4,10,11].

Due to the high rates of postural and balance deficits present following concussive injury, postural stability assessments are an important component of the concussion evaluation [4,12,13]. The Balance Error Scoring System (BESS) was originally developed as an easily administered, cost-effective assessment tool and is commonly used by healthcare providers for the evaluation of postural stability before and after concussion. The BESS consists of three stances: double-leg stance (feet together), single-leg stance (standing on the nondominant leg) and a tandem stance (nondominant foot behind the dominant foot in a heel-to-toe fashion). The stances are performed with the eyes closed and the hands on the hips, first on a firm surface and then on a foam surface. Error scores are counted for each condition. The aim of these stances is to isolate each component of the balance system [14]. A modified version of the BESS (mBESS) consists of testing the three stances on a firm surface only. The mBESS is the recommended postural control test of the 4th International Consensus Statement on Concussion in Sport and has been incorporated into the widely-used Sport Concussion Assessment Tool 3 (SCAT3) [15]. Additionally, in this 3rd edition of the SCAT, the more recently developed timed tandem gait has also been added as a supplement assessment of balance [15].

Despite the incorporation of the BESS and mBESS into many concussion assessment protocols, studies have emphasized their limitations, including insufficient repeatability, poor reliability, fatigue effects, influences from musculoskeletal injuries and learning effects [16,17]. The reliability of the BESS ranges from poor to good, with some studies reporting reliability coefficients below clinically acceptable levels [18–20]. The poor reliability of the BESS is compounded by the highly subjective nature of the scoring system resulting in poor inter-rater reliability [21].

Given the difficult task of identifying subtle balance anomalies, as well as correctly quantifying errors on the BESS, a more objective balance test is needed. The purpose of this study was to investigate preseason baseline mBESS performance in a collegiate football cohort and compare these scores to the Balance Test by King–Devick, a new objective mobile balance measurement tool.

## Materials & methods

In total, 82 collegiate football players between the ages of 18 and 22 years were recruited for participation in this study. After obtaining informed written consent, baseline mBESS examinations were performed by the study team during the athletes’ preseason physical evaluations. The study team consisted of neurologists trained in the baseline assessment for concussion. All testing was completed in the training room with multiple athletes tested simultaneously. Study protocols were approved by the Mayo Clinic Institutional Review Board.

Testing procedures, including error definitions and scoring, were obtained directly from the SCAT3 procedural instructions [15]. Subjects were informed that the testing involved completing three, 20-s stances (double leg, single leg, tandem) and that their errors in balance would be counted. Each stance was defined as the following: double-leg stance (DS; feet together), single-leg stance (SS; standing on the nondominant foot, dominant foot at approximately 30-degrees of hip flexion and 45-degrees of knee flexion) and tandem stance (TS; standing heel-to-toe with the nondominant foot in back). Athletes were asked to position themselves in each of these stances with their hands placed on the hips at the level of the iliac crest, and to maintain this posture for 20 s. Errors were defined as any of the following:

- Lifting the hands off of the iliac crest;
- Opening the eyes;
- Any step, stumble or fall;
- Moving the hip into >30-degrees abduction;
- Lifting the forefoot or heel;
- Remaining out of test position >5 s.

The 20-s trial began when the athlete was in the proper stance position with their eyes closed. Also, as per SCAT3 instructions, the maximum total errors for each 20-s condition was ten, with a maximum error score given for athletes that could not maintain position for a minimum of 5 s for each individual stance. If an athlete committed multiple errors simultaneously, only one error was recorded. Each mBESS was performed on a firm surface in the athletic training room without shoes [15,22]. Testing was performed prior to the start of the season, and before the start of any full-contact practice sessions. None of the athletes presented with symptomatic effects of acute concussion or postconcussion syndrome. MBESS performance during this study was considered to be each athlete’s preseason balance baseline.

**Figure 1.**
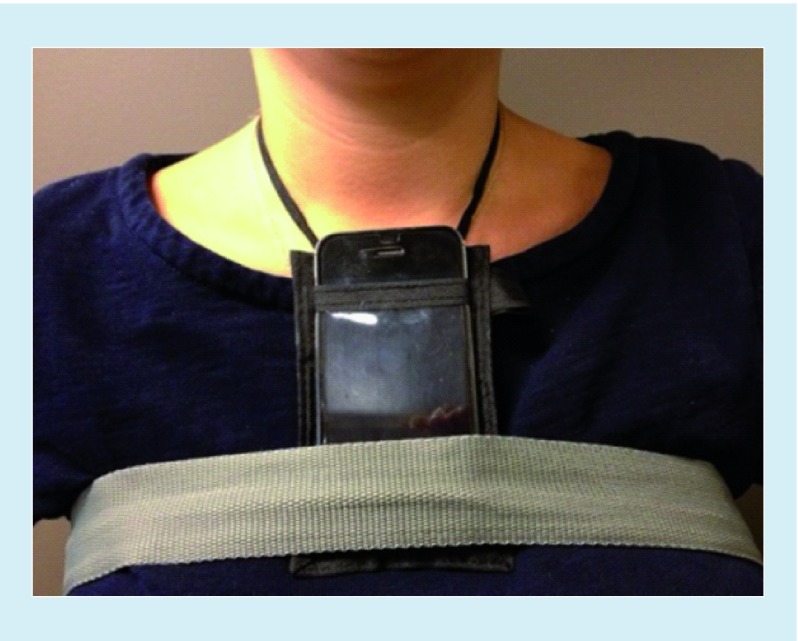
**The device is placed into a holder around the neck, the holder is adjusted to bring the device just below the neck and is then secured to the body with a chest strap.**

**Figure 2.**
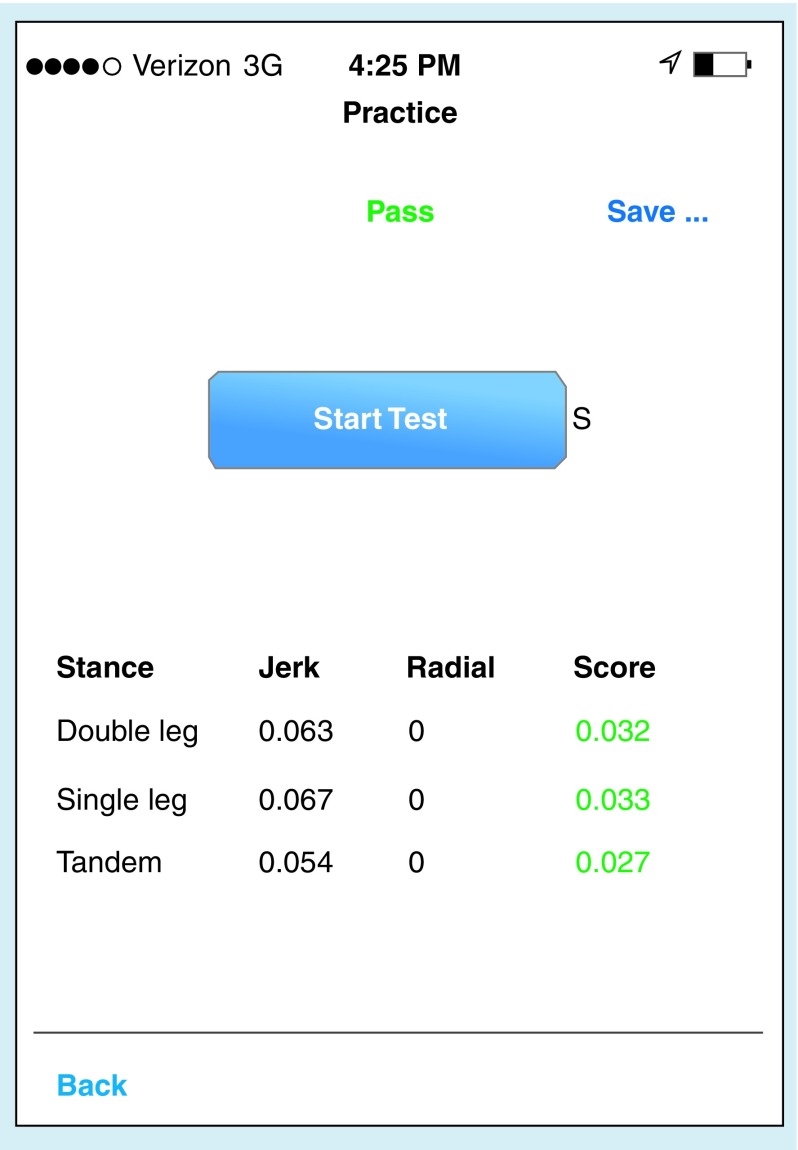
**Screen shot of the balance test application.**

Simultaneous with mBESS evaluation, quantitative measurements of balance and postural stability were obtained by using the Balance Test by King–Devick, a propriety software (King–Devick Test Inc., IL, USA) that utilizes tri-axial coordinate data from the internal accelerometers of mobile devices to determine a single quantitative and objective balance score through measurements of pitch, yaw and roll. The Balance Test by King–Devick (v0.80) is an application which was utilized on an Apple iPhone mobile device (Apple Inc., CA, USA) secured directly below each athlete’s neck (Figure 1). This location has been shown to be ideal for measurement of maximum postural sway [23]. Balance data were collected during each 20-s mBESS condition (DS, SS, TS). Deflections of the accelerometer were recorded by the device software during the trials and were used to calculate a Jerk Score based on changes in acceleration over time. A Radial Distance Score was calculated using average velocity measurements over time and determined across the x-, y- and z-axes. A final composite balance score for each stance is determined by an average of the Jerk Score and Radial Distance (Figure 2). The device instructs the examiner step-by-step through the test protocol in order to maintain consistency between test administrations.

### Statistical analysis

Data analyses were performed using Stata 12.0 (StataCorp, TX, USA). Descriptive statistics were used to determine mean mBESS and Balance Test scores for the cohort. Differences in mBESS and Composite Balance Test stance scores were calculated and compared using analysis of variance (ANOVA) test with Tukey *post hoc* comparisons. Associations between mBESS and Composite Balance Test scores, as well as mBESS and history of prior concussions, were calculated using Pearson’s correlation coefficient. For all statistical tests, significance was set *a priori* at p < 0.05.

## Results

The characteristics of all 82 athletes are summarized in Table 1. All athletes were male football players who represented a wide range of player positions.

**Table 1.** **Characteristics of the collegiate football cohort in this study.**

**Athlete demographics**	**All athletes (n = 82)**
Age, mean ± SD, range	19.9 ± 1.5, 18–22 years

Gender, male, n (%)	82 (100)

Prior concussions, self-report, n (%)	35 (42.7)

Number of concussions, mean ± SD, range	0.76 ± 1.35, 0–10

Position, n (%):	
• Defensive line	14 (17.1)
• Offensive line	13 (15.9)
• Wide receiver	10 (12.2)
• Linebacker	10 (12.2)
• Cornerback	7 (8.5)
• Safety	7 (8.5)
• Running back	5 (6.1)
• Quarterback	4 (4.9)
• Tight end	4 (4.9)
• Fullback	3 (3.7)
• Long snapper	2 (2.4)
• Kicker	2 (2.4)
• Defensive back	1 (1.2)

**Figure 3.**
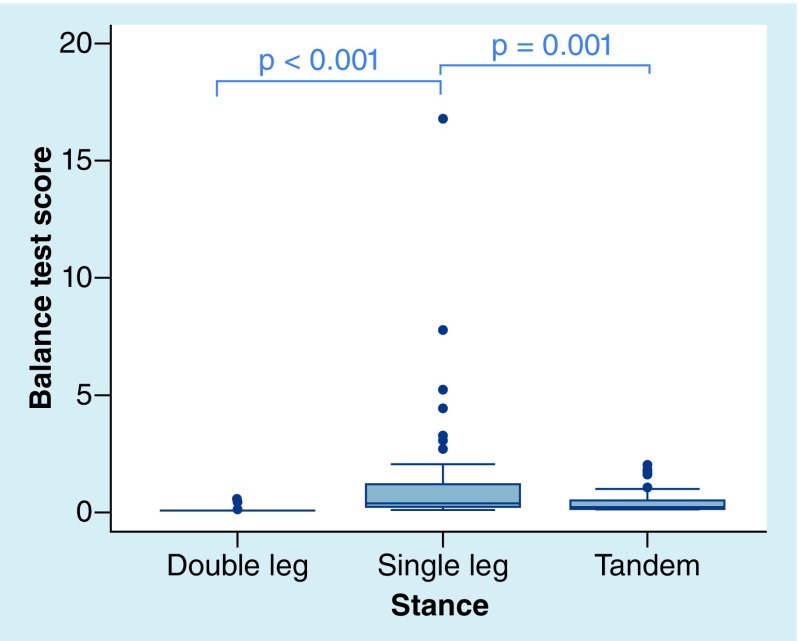
**Distributions of King–Devick balance test scores by stance.** The line within the box defines the median value. The range of the box corresponds to the interquartile range (25th and 75th percentile). Whiskers extending from the box plot represent the range of observations, excluding outliers. The small circles beyond the whiskers represent the outliers. Analysis demonstrated a significant effect of score by stance (F [2, 243] = 13.23, p < 0.001, one-way ANOVA).

**Figure 4.**
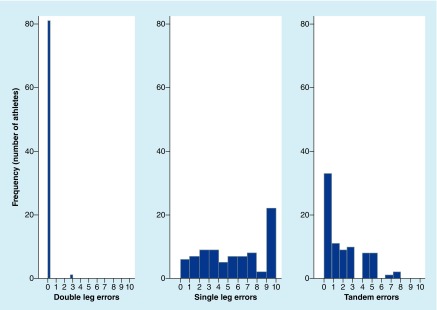
**Frequency of error scores by stance.**

**Figure 5.**
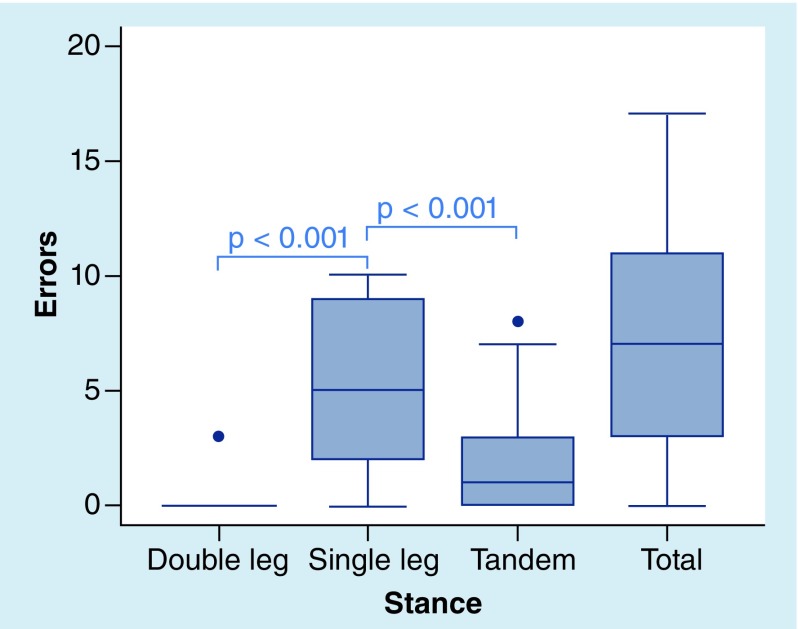
**Box plots show the distribution of modified balance error scoring system scores by stance and total modified balance error scoring system score.** The line within the box defines the median value. The range of the box corresponds to the interquartile range (25th and 75th percentile). Whiskers extending from the box plot represent the range of observations, excluding outliers. The small circles beyond the whiskers represent the outliers. Analysis demonstrated a significant effect of stance (F [2, 243] = 110.2, p < 0.001, one-way ANOVA).

The cohort’s mBESS stance mean scores and total scores are provided in Table 2. The mean summed error score including all three stances (total error score) was 7.23 ± 4.65. A one-way analysis of variance (ANOVA) was calculated between the three stances. The analysis was significant, F(2, 243) = 110.2, p < 0.001. The mean error score for SS was significantly higher than the TS and DS conditions (5.32 SS vs 1.88 TS, t[243] = -9.52, r = 0.52, p < 0.001; 0.04 DS, t[243] = 14.62, r = 0.68, p < 0.001). There were 593 total errors committed by the cohort. The SS condition accounted for 74% of these errors (436 errors) as compared with 26% (154 errors) during TS and less than 1% (3 errors) during DS conditions. The mBESS error score distributions show high variability of scores, particularly for SS (Figures 3 & 4). At baseline, 21% of the cohort (17 athletes) had the maximum error score in SS (10 errors), while there were no athletes with a maximum error score for TS or DS conditions. Thirty-eight percent (31 athletes) had a SS score of seven or higher, while only 4% (3 athletes) had a TS score of seven or higher. No athlete had a score greater than three in DS (Figure 5). Of note, there were no significant associations between self-reported concussion history and total mBESS score (r(76) = 0.02, p = 0.84, Pearson correlation coefficient), nor between self-reported concussions and mBESS subtests (DS: r(76) = -0.06, p = 0.58; SS: r(76) = 0.13, p = 0.25; TS: r(76) = -0.15, p = 0.18).

**Table 2.** **Modified Balance Error Scoring System scores by stance.**

**Test**	**Mean**	**SD**	**Total errors**
Double leg	0.04	0.33	3

Single leg	5.32	3.40	436

Tandem	1.88	2.09	154

Total	7.23	4.65	593

The mean Balance Test scores by stance are presented in Table 3. A one-way analysis of variance (ANOVA) was calculated between the three stances. The analysis was significant, F(2, 243) = 13.23, p < 0.001. Similar to mBESS results, the SS score was significantly higher than DS (1.06 vs 0.07, t[243] = 5.01, r = 0.31, p < 0.001) and TS (1.06 vs. 0.37, t[243] = -3.51, r = 0.19, p < 0.001). Figure 3 provides the variability of Balance Test score distributions by stance.

**Table 3.** **King–Devick Balance Test scores by stance.**

**Test**	**Mean**	**SD**
Double leg	0.07	0.06

Single leg	1.06	2.14

Tandem	0.37	0.40

There was no significant correlation between the subjective mBESS and objective Balance Test measures for DS (r[76] = 0.03, p = 0.80) and TS (r[76] = 0.18, p = 0.10). There were significant, but weak correlations for balance scores between the mBESS and the Balance Test for SS (r[76] = 0.25, p = 0.03).

## Discussion

Imbalance is among the most commonly reported subjective symptoms after concussion and among the most commonly reported findings in concussed athletes undergoing objective after-injury testing using force plate technologies. As many athletes under-report or fail to report concussion symptoms, objective measures of deviation from baseline performance on examinations of balance are one of many tools useful in making informed remove-from-play decisions. These results are also important assessments as part of a comprehensive protocol for making return-to-play decisions.

A key finding in this study is the high degree of variability that existed during the SS subtest of the mBESS among this cohort of nonconcussed, elite athletes at their asymptomatic baseline. This high degree of variability combined with a large proportion of athletes presenting with the maximum number of errors at baseline raises concerns over the utility of the SS condition in identifying abnormalities after suspected concussion. Additionally, the highly subjective scoring system, the poor inter-rater reliability of the mBESS [18–20] and the fact that the mBESS may be difficult to perform in the presence of musculoskeletal injuries (e.g., knee or ankle injuries) [19] suggest that it may not be an appropriate tool for establishing baseline balance performance, after-injury decrement from baseline and fitness for return-to-play.

In many instances, the rapid identification of a deviation from baseline is critical in athletes under evaluation for suspected concussion. These data also help to support the conclusion that the SS condition may be effectively eliminated from the standard mBESS protocol as the DS and TS conditions may be more effective at identifying abnormalities obtained due to concussion. The mBESS also assumes that each error is of identical weighting in computing the overall score. Our data suggest that some error elements may not correlate well with true, objective postural stability.

Minimizing the inherent inter-rater variability and subjective scoring of the mBESS should allow for the establishment of baseline performance, in addition to improved detection of balance deficits after concussion, particularly when assessed by novice sideline screeners. An objective, easily reproducible protocol for assessing baseline and after-injury balance may serve to improve accuracy when making decisions to remove a player after a suspected concussion. In this study, preseason mBESS scores for DS and TS conditions were consistent with previously reported literature, which demonstrated low variance associated with the DS condition [4,13,24–27].

The mBESS errors and Balance Test scores for the SS were surprisingly high at preseason testing, with many subjects obtaining maximum scores on mBESS in the absence of acute concussion or postconcussion syndrome. This finding is consistent with previous data [19], and is likely due to a ceiling effect of the mBESS (maximum score = 10 per condition). Objective measures of postural stability, such as the King–Devick Balance Test, may help address the limitations of the mBESS through the calculation of an objective score (with no maximum score), while simultaneously addressing the inconsistencies associated with the subjective nature of scoring and poor inter-rater reliability. However, worse scores, both subjective and objective, may be compounded by the apparent difficulty in performing the SS condition even in nonconcussed athletes.

Our data indicate poor correlation between mBESS errors and K-D Balance Test scores at baseline. We believe this may be due to a number of reasons. First the ceiling effect of the maximum ten errors for each stance of the mBESS limits our ability to determine correlations for athletes with high (worse) scores of balance. Second, each error counted during mBESS testing may not have necessarily been associated with changes in postural movement. For example errors due to opening of eyes or lifting hands off of the iliac crest could have occurred in the absence of any movement that would have translated to the accelerometer positioned on the athletes’ chest. Despite this lack of correlation, incorporating an objective balance tool such as the K-D Balance Test may supplement balance assessment in the setting of concussion. Further research is necessary to thoroughly examine the ability of the K-D Balance Test to detect changes in balance performance associated with concussion.

There are several limitations in this study. All participants were male football players aged 18–22 years. Gender and age play significant roles in balance function [28,29]. Additional evaluation on athletes with a broad age range, as well as including a female cohort, will be needed to address this limitation. Although 43% (n = 35) of athletes reported prior concussions, none endorsed symptoms of acute concussion or postconcussion syndrome at baseline based on the current gold standard – a clinical evaluation by the team physician and a neurologist. The authors recognize that there are uncertainties that exist when relying upon symptom checklists and self-reporting by individuals. The medical team concluded that each athlete was at his baseline in the context of acceptable performance on other preseason objective evaluations. These included: neurologic history and performance on the computerized Immediate Post-concussion Assessment and Cognitive Test (ImPACT), Standardized Assessment of Concussion (SAC) and King–Devick Test (rapid number naming test). However, without an objective biomarker, it is difficult to determine with certainty whether an individual’s performance on the mBESS during this study was reflective of lingering deficits from prior concussions. Further evaluation should include data from a wide demographic to better establish normative values for both genders, additional age groups and athletes in sports considered to be low-risk for concussion. In future studies, our group aims to assess the accuracy and reliability of the Balance Test by King–Devick in identifying a significant deviation from baseline balance performance during sideline assessments for concussion at the time of injury as well as comparisons in performance of nonconcussed, control athletes. These data will also serve to improve our understanding of how sideline accelerometry measures may be used to identify and document balance dysfunction.

## Conclusion

Our data show that single leg stance performance is highly variable at baseline, even in the absence of concussion in collegiate athletes, and therefore may have limited use in balance evaluation to detect changes secondary to concussion.

## Future perspective

As public awareness of sports-related concussion continues to grow, future study will be driven to further investigate the utility of tools for both the sideline evaluation and clinical management of concussion. Similarly, expanding knowledge of the pathophysiology of concussive injury, recovery and healing as well as factors that put certain individuals at higher risk for injury and prolonged sequelae will give all stakeholders involved improved understanding of concussion leading to enhanced future management of sports-related concussion.

**Executive summary**

- This was a study of balance performance in elite level, college football players at preseason baseline using the modified Balance Error Scoring System (mBESS), the balance component of the Sport Concussion Assessment Tool 3rd Edition (SCAT3) and the Balance Test by King–Devick, an objective, mobile software utilizing mobile device accelerometer data to measure postural balance.
- Balance measures were completed during three stances (single leg, double leg and tandem) for 20 s.
- Approximately 74% of mBESS errors (total of 593 errors) at baseline were committed while performing the single leg stance.
- Approximately 21% of athletes in the cohort exhibited the maximum error score (ten errors) on single leg stance and 38% of athletes had a single leg stance score of greater than seven errors.
- Results from this study indicate a high degree of variability during the single leg stance subtest of the mBESS baseline testing. As such, detecting change in performance in mBESS, particularly when using the single leg stance, is difficult and may have limited utility in identifying abnormalities associated with concussion.
- Approximately 42.7% of athletes self-reported having sustained a prior concussion.
- There were no correlations between self-reported concussion history and total mBESS scores.
- Future studies will determine the utility of accelerometry tools, such as the Balance Test, on the sidelines in identifying balance dysfunctions associated with concussion injury.

## References

[1] Rutland-Brown W, Langlois JA, Thomas KE, Xi YL (2006). Incidence of traumatic brain injury in the United States, 2003. *J. Head Trauma Rehabil.*.

[2] Collins MW, Kontos AP, Reynolds E, Murawski CD, Fu FH (2014). A comprehensive, targeted approach to the clinical care of athletes following sport-related concussion. *Knee Surg. Sports Traumatol. Arthrosc.*.

[3] Zylka J, Lach U, Rutkowska I (2013). Functional balance assessment with pediatric balance scale in girls with visual impairment. *Pediatr. Phys. Ther.*.

[4] Guskiewicz KM, Ross SE, Marshall SW (2001). Postural Stability and Neuropsychological Deficits After Concussion in Collegiate Athletes. *J. Athl. Train.*.

[5] Maskell F, Chiarelli P, Isles R (2006). Dizziness after traumatic brain injury: overview and measurement in the clinical setting. *Brain Inj.*.

[6] Reddy CC, Collins MW, Gioia GA (2008). Adolescent sports concussion. *Phys. Med. Rehabil. Clin. N. Am.*.

[7] Cutrer FM, Baloh RW (1992). Migraine-associated dizziness. *Headache*.

[8] Furman JM, Marcus DA, Balaban CD (2003). Migrainous vertigo: development of a pathogenetic model and structured diagnostic interview. *Curr. Opin. Neurol.*.

[9] Savundra PA, Carroll JD, Davies RA, Luxon LM (1997). Migraine-associated vertigo. *Cephalalgia*.

[10] Geurts AC, Ribbers GM, Knoop JA, Van Limbeek J (1996). Identification of static and dynamic postural instability following traumatic brain injury. *Arch. Phys. Med. Rehabil.*.

[11] Rubin AM, Woolley SM, Dailey VM, Goebel JA (1995). Postural stability following mild head or whiplash injuries. *Am. J. Otol.*.

[12] Ingersoll CD, Armstrong CW (1992). The effects of closed-head injury on postural sway. *Med. Sci. Sports Exerc.*.

[13] Riemann BL, Guskiewicz KM (2000). Effects of mild head injury on postural stability as measured through clinical balance testing. *J. Athl. Train.*.

[14] Guskiewicz KM (2003). Assessment of postural stability following sport-related concussion. *Curr. Sports Med. Rep.*.

[15] Mccrory P, Meeuwisse WH, Aubry M (2013). Consensus statement on concussion in sport: the 4th International Conference on Concussion in Sport held in Zurich, November 2012. *Br. J. Sports Med.*.

[16] Fox ZG, Mihalik JP, Blackburn JT, Battaglini CL, Guskiewicz KM (2008). Return of postural control to baseline after anaerobic and aerobic exercise protocols. *J. Athl. Train.*.

[17] Wilkins JC, Valovich McLeod TC, Perrin DH, Gansneder BM (2004). Performance on the Balance Error Scoring System decreases after fatigue. *J. Athl. Train.*.

[18] Portney LG, Watkins MP (2009). *Foundations of Clinical Research: Applications to Practice.*.

[19] Hunt TN, Ferrara MS, Bornstein RA, Baumgartner TA (2009). The reliability of the modified Balance Error Scoring System. *Clin. J. Sport Med.*.

[20] Finnoff JT, Peterson VJ, Hollman JH, Smith J (2009). Intrarater and interrater reliability of the Balance Error Scoring System (BESS). *PM R*.

[21] Bell DR, Guskiewicz KM, Clark MA, Padua DA (2011). Systematic review of the Balance Error Scoring System. *Sports Health*.

[22] Guskiewicz KM (2011). Balance assessment in the management of sport-related concussion. *Clin. Sports Med.*.

[23] Brown HJ, Siegmund GP, Guskiewicz KM, Van Den Doel K, Cretu E, Blouin JS (2014). Development and validation of an objective Balance Error Scoring System. *Med. Sci. Sports Exerc.*.

[24] Reimann BL, Guskiewicz KM, Shields EW (1999). Relationship between clinical and forceplate measures of postural stability. *J. Sport Rehabil.*.

[25] Valovich TC, Perrin DH, Gansneder BM (2003). Repeat administration elicits a practice effect with the Balance Error Scoring System but not with the standardized assessment of concussion in high school athletes. *J. Athl. Train.*.

[26] Valovich Mcleod TC, Perrin DH, Guskiewicz KM, Shultz SJ, Diamond R, Gansneder BM (2004). Serial administration of clinical concussion assessments and learning effects in healthy young athletes. *Clin. J. Sport Med.*.

[27] Susco TM, Valovich Mcleod TC, Gansneder BM, Shultz SJ (2004). Balance recovers within 20 minutes after exertion as measured by the Measured by the Balance Error Scoring System. *J. Athl. Train.*.

[28] Valente M (2007). Maturational effects of the vestibular system: a study of rotary chair, computerized dynamic posturography, and vestibular evoked myogenic potentials with children. *J. Am. Acad. Audiol.*.

[29] Shimizu K, Asai M, Takata S, Watanabe A (3–7 October 1994). The development of equilibrium function in childhood. *12th International Symposium on Posture and Gait.*.

